# Functional Characterization of 8-Oxoguanine DNA Glycosylase of *Trypanosoma cruzi*

**DOI:** 10.1371/journal.pone.0042484

**Published:** 2012-08-02

**Authors:** Carolina Furtado, Marianna Kunrath-Lima, Matheus Andrade Rajão, Isabela Cecília Mendes, Michelle Barbi de Moura, Priscila Carneiro Campos, Andrea Mara Macedo, Glória Regina Franco, Sérgio Danilo Junho Pena, Santuza Maria Ribeiro Teixeira, Bennett Van Houten, Carlos Renato Machado

**Affiliations:** 1 Departamento de Bioquímica e Imunologia, Instituto de Ciências Biológicas, Universidade Federal de Minas Gerais, Belo Horizonte, Minas Gerais, Brazil; 2 Department of Pharmacology and Chemical Biology, University of Pittsburgh School of Medicine and the University of Pittsburgh Cancer Institute, Hillman Cancer Center, Pittsburgh, Pennsylvania, United States of America; Saint Louis University, United States of America

## Abstract

The oxidative lesion 8-oxoguanine (8-oxoG) is removed during base excision repair by the 8-oxoguanine DNA glycosylase 1 (Ogg1). This lesion can erroneously pair with adenine, and the excision of this damaged base by Ogg1 enables the insertion of a guanine and prevents DNA mutation. In this report, we identified and characterized Ogg1 from the protozoan parasite *Trypanosoma cruzi* (TcOgg1), the causative agent of Chagas disease. Like most living organisms, *T. cruzi* is susceptible to oxidative stress, hence DNA repair is essential for its survival and improvement of infection. We verified that the *TcOGG1* gene encodes an 8-oxoG DNA glycosylase by complementing an Ogg1-defective *Saccharomyces cerevisiae* strain. Heterologous expression of *TcOGG1* reestablished the mutation frequency of the yeast mutant *ogg1^−^/^−^* (CD138) to wild type levels. We also demonstrate that the overexpression of *TcOGG1* increases *T. cruzi* sensitivity to hydrogen peroxide (H_2_O_2_). Analysis of DNA lesions using quantitative PCR suggests that the increased susceptibility to H_2_O_2_ of *TcOGG1*-overexpressor could be a consequence of uncoupled BER in abasic sites and/or strand breaks generated after TcOgg1 removes 8-oxoG, which are not rapidly repaired by the subsequent BER enzymes. This hypothesis is supported by the observation that *TcOGG1*-overexpressors have reduced levels of 8-oxoG both in the nucleus and in the parasite mitochondrion. The localization of TcOgg1 was examined in parasite transfected with a TcOgg1-GFP fusion, which confirmed that this enzyme is in both organelles. Taken together, our data indicate that *T. cruzi* has a functional Ogg1 ortholog that participates in nuclear and mitochondrial BER.

## Introduction

*Trypanosoma cruzi* (*T. cruzi*) is the causative agent of Chagas disease, a debilitating illness that afflicts about 8–10 million people in Latin America where it has a considerable economic and social impact [1]. This protozoan belongs to the order Kinetoplastida, which includes unicellular flagellated organisms that are characterized by the presence of the kinetoplast, a DNA-containing granule localized within their single mitochondrion [2].

*T. cruzi* presents a complex life cycle that requires both invertebrate and mammalian hosts. The parasite undergoes extracellular multiplication in the insect vector, but grows by obligate intracellular multiplication cycles in vertebrate hosts [3]. Therefore, *T. cruzi* needs to deal with the oxidative burst from the hosts immune systems, which results in the production of superoxide anion radicals (O_2_^.^) and subsequent other reactive oxygen species (ROS) such as hydrogen peroxide [4].

Excess ROS could have deleterious effects to cells since these agents can oxidize several molecules such as lipids, carbohydrates, proteins and nucleic acids [5]. In DNA, the action of ROS can cause single- and double-strand breaks (SSBs and DSBs, respectively), base loss and base oxidation. Among the large variety of oxidative modifications that can occur in DNA, 8-oxoguanine (8-oxoG) represents one of the most abundant and best characterized lesions. The biologic importance of 8-oxoG is due to its propensity to mispair with adenine residues, leading to an increased frequency of spontaneous G:C→T:A mutations. It is estimated that the steady-state level of this lesion in human cells is about 10^3^/day [5].

It is generally assumed that oxidative DNA lesions are usually dealt with by base excision repair (BER) pathway. This multistep repair pathway is initiated by a specific DNA glycosylase that recognizes and removes the modified base, leaving an abasic site (AP site) that is potentially cytotoxic and mutagenic. Subsequently, the DNA backbone is cleaved by an AP endonuclease and the repair is completed by the activity of a phosphodiesterase, a DNA polymerase and a DNA ligase [6].

The 8-oxoG repair is also part of a multi-defense mechanism, the so-called GO system, which comprises three enzymes in eukaryotes: the glycosylases Ogg1 and MYH (MutY homologue), and the hydrolase MTH (MutT homologue). Ogg1 prevents mutagenesis by the removal of 8-oxoG from the 8-oxoG:C pair. On the other hand, MYH performs the excision of adenine from the 8-oxoG:A pair in DNA. The hydrolase MTH inhibits the incorporation of the oxidized guanine into DNA through hydrolysis of 8-oxo-dGTP to 8-oxo-dGMP [7], [8].

Ogg1 is a bifunctional glycosylase since it also has an associated lyase activity, which can attack the abasic site after the removal of the 8-oxodG base. This enzyme acts both in the nucleus and the mitochondria [9]. Different Ogg1 polymorphisms are described as being involved in numerous diseases, such as several forms of cancer, diabetes and Huntington disease [10], [11], [12], [13], [14], [15]. Ogg1 has been characterized in several eukaryotes from simpler organisms, as *Saccharomyces cerevisiae*, to more complex species, as *Arabdopsis thaliana* and *Homo sapiens* [9], [16], [17], [18], [19]. *In silico* analysis of the *T. cruzi* genome showed that this protozoan presents one putative copy of the *OGG1* gene [20], [21]. Given the importance of Ogg1 in preventing oxidative stress-induced mutagenesis, we investigated the role of this *T. cruzi* gene by complementing *OGG1*-deficient yeast and by studying the phenotype of over-expressing *TcOGG1* in epimastigotes analyzing nuclear and mitochondrial DNA lesions after oxidative treatment.

## Results

### *Trypanosoma cruzi* has a putative OGG1 orthologue

**Figure 1:**
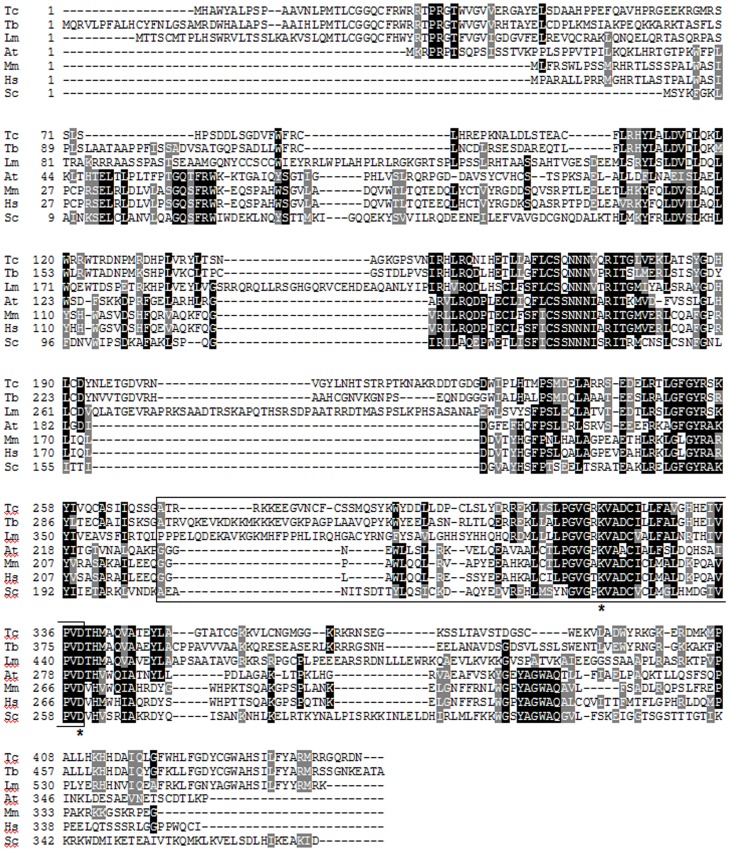
Alignments with the predicted *TcOGG1* products. Amino acid sequence comparison of the predicted product of the *OGG1* gene from *Trypanosoma cruzi* (*TcOGG1*_B; Tc), *Trypanosoma brucei* (Tb), *Leishmania major* (Lm), *Arabidopsis thaliana* (At), *Mus musculus* (Mm), *Homo sapiens* (Hs) and *Saccharomyces cerevisiae* (Sc). Residues shaded in black indicate identical amino acids. Residues shaded in gray are functionally similar. Residues enclosed by the box belong to the HhH-G/PD motif. Asterisks correspond to the Ogg1 catalytic lysine residue and its auxiliary aspartic acid.

The sequencing of *T. cruzi* genome showed that this protozoan has a putative 8-oxoguanine DNA glycosylase gene (*TcOGG1*), present in single copy in the CL Brener strain [20]. Fig. 1 shows the alignment of the *TcOGG1*-deduced amino acid sequence and several orthologs from other organisms. Sequence analysis revealed that TcOGG1 is a 449 amino acid protein that exhibits a helix-hairpin-helix followed by a Gly/Pro-rich loop and a conserved aspartic acid (HhH-G/PD motif). This domain is the hallmark of the base excision DNA repair HhH-G/PD protein superfamily, in which Ogg1 is included, and contains essential amino acids for catalysis and substrate recognition [22], [23], [24]. Moreover, TcOgg1 has the Ogg1 catalytic lysine residue and its auxiliary aspartic acid, present in the positions 319 and 338, respectively.

Analysis of the *T. cruzi* genome database (http://www.genedb.org) also showed that the CL Brener strain is heterozygote for *OGG1* gene. The two alleles (named here as *TcOGG1*_A and *TcOGG1*_B) display 13 amino acids substitutions, two of them being synonymous substitutions (Fig. S1). These differences do not occur in essential regions for Ogg1 activity, suggesting that the proteins encoded by these two alleles do not possess major functional biological divergences. In addition, both alleles carry putative targeting sequences to the nucleus and mitochondria (Fig. S1).

### *TcOGG1* complements *ogg1^−^/^−^* yeast

In order to investigate the activity of TcOgg1 *in vivo*, we examined its ability to complement the hypermutator phenotype of an *ogg1* ^−^/^−^ yeast strain [25]. We used *Saccharomyces cerevisiae* as a heterologous system for functional studies of the *TcOGG1* gene due to the toxicity observed when we expressed this gene in *Escherichia coli* (Fig. S2 A–C).

**Figure 2:**
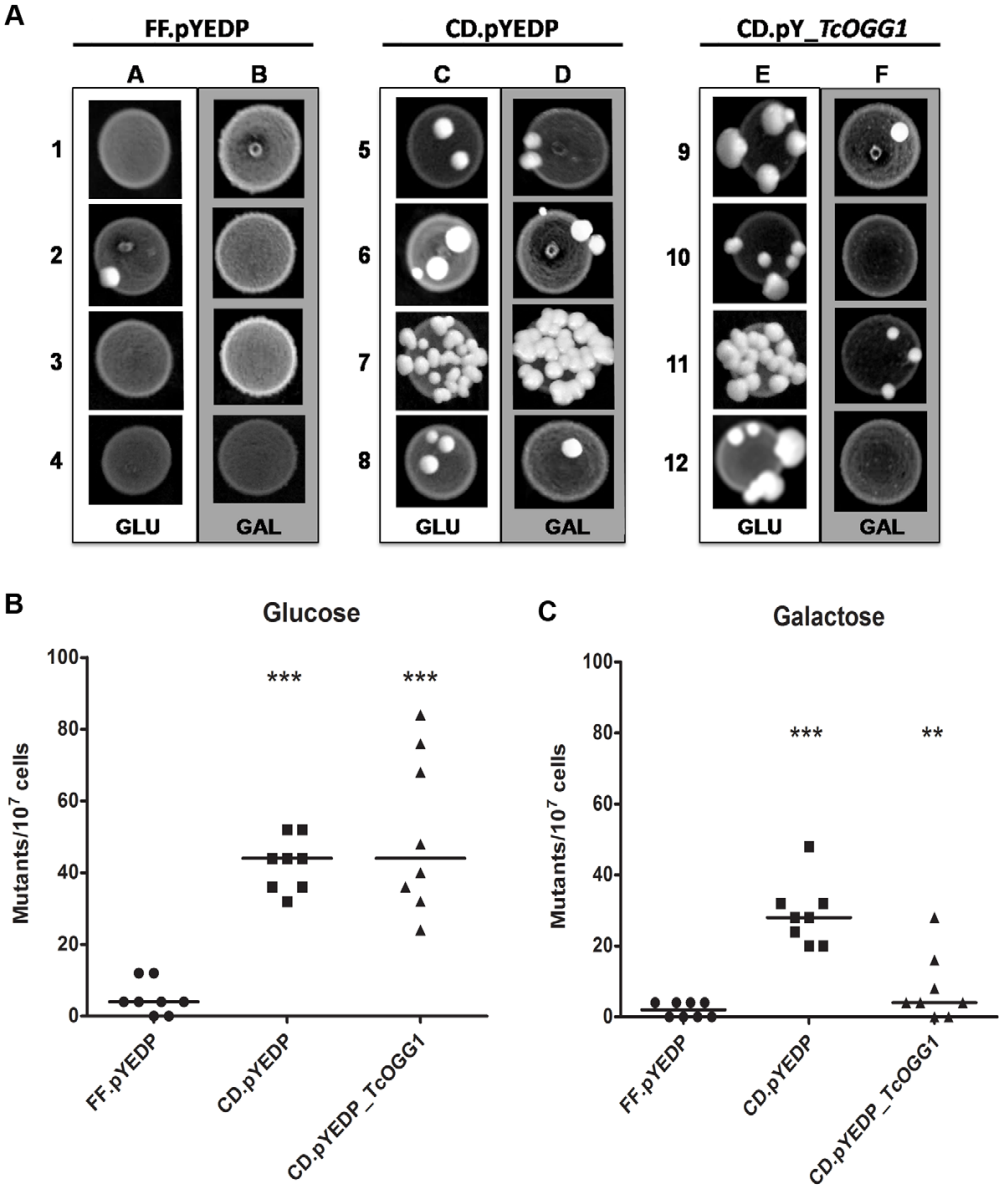
Heterologous complementation assay with FF18733 (WT) and CD138 (*ogg1*-) yeast. **A**) Qualitative analysis. Cells were transformed with pYEDP (WT (FF.pYEDP) and o*gg1*- (CD.pYEDP)) or pYEDP_*TcOGG1* (only *ogg1*- (CD.pY_*TcOGG1*)). Yeasts were grown in plates containing glucose (GLU; without expression of the gene inserted in the vector) or galactose (GAL; expression of *TcOGG1*, due to galactose promoter), without lysine (selection of Lys^+^ mutants). Letters refer to growth on glucose (A, C and E) or galactose (B, D and F). Numbers refer to different clones. **B and C**) Quantitative analysis. Mutants obtained in the assay showed in Fig. 2A were counted, originating Figures 2B–C. Fig. 2B shows the results for glucose, whereas Fig. 2C displays the results for galactose. The graphics were plotted using median and the statistical analysis used was Kruskal-Wallis test (One way ANOVA). FF.pYEDP (•); CD.pYEDP (▪); CD.pYEDP_*TcOGG1* (▴). ***- P value<0,001; ** - P value<0,01.

After cloning both alleles (*TcOGG1*_A and *TcOGG1*_B) independently in a galactose-inducible yeast vector, we transformed wild type FF18733 [26] and *OGG1*-deficient CD138 [25] yeast strains with these constructs. The spontaneous mutation frequency of the referred strains was assessed by determining the number of Lys^+^ revertants in *S. cerevisiae* cultures that were grown in plates containing glucose or galactose. The results seen in Fig. 2A–C show that the mutation frequency was higher (p<0.001) in *OGG1*-deficient cells when compared to wild type cells (Fig. 2A, columns A/C and Fig. 2B). As expected, in the presence of glucose, *ogg1^−^/^−^* cells carrying *TcOGG1*_B behaved as those carrying the empty vector (Fig. 2A, column E/C and Fig. 2B). As shown in figures 2A and C, galactose-induced expression of *TcOGG1*_B in *OGG1^−^/^−^* cells reduced the number of spontaneous mutants to wild type levels. This revertant phenotype presented by yeast cells expressing *TcOGG1*_A was similar in cells expressing *TcOGG1*_B (Fig. S3 A–B). Therefore, these data indicate that the minor sequence differences between the two alleles do not result in differences in their biological role.

### Overexpression of *TcOGG1* sensitizes *T. cruzi* to H_2_O_2_

**Figure 3:**
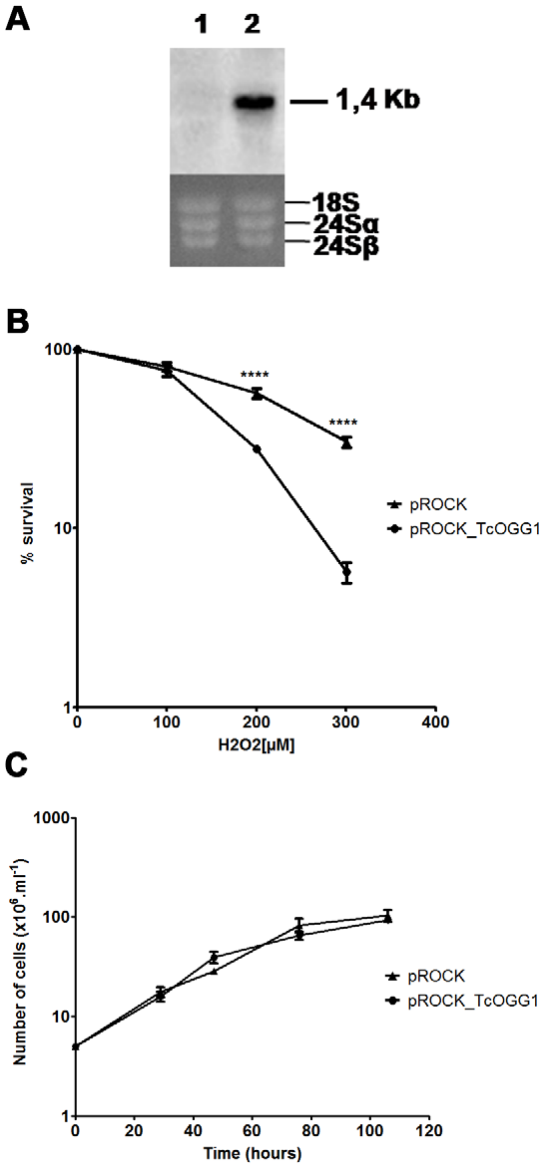
H_2_O_2_ treatment of *TcOGG1*-overexpressor *T. cruzi*. **A**) **Northern blot analyses of CL Brener strain transfected with empty vector (1) or with pROCK_** ***TcOGG1*** **(2).** Total RNA was extracted and probed with *TcOGG1* DNA. The agarose gel stained with ethidium bromide shows total RNA extracted from these parasites. **B**) ***T. cruzi OGG1*** **-overexpressor survival curve after H_2_O_2_ treatment.** Parasites were treated with different H_2_O_2_ doses and after 3 days were counted. Survival percentage was measured in relation to untreated cells. **C**) ***T. cruzi OGG1*** **-overexpressor growth curve.** Cells were counted in certain time intervals through a period of approximately 100 hours. The curves are the average of three independent experiments, each one in triplicate. Bars represent SEM. Statistical analysis used was unpaired *t* test. CL Brener strain transfected with pROCK (▴) or with pROCK_*TcOGG1* (•). **** - P value<0,0001.

We generated a *T. cruzi* population stably overexpressing *TcOGG1* using the integrative vector pROCK [27], [28], [29], [30], [31] carrying the gene *TcOGG1*, and analyzed the response of *TcOGG1*-overexpressing cells to hydrogen peroxide treatment. This substance causes oxidative DNA lesions, including 8-oxoG, which is recognized and excised by Ogg1. Control parasites transfected with the empty vector showed undetectable levels of TcOGG1 mRNA, but on the other hand, transgenic parasites that has integrated the pROCK-*TcOGG1* vector in its genome showed high levels of a 1,4 kb mRNA corresponding to the transfected copy of *TcOGG1* (Fig. 3A). As shown in Fig. 3B, *TcOGG1* overexpression considerably decreased the survival of *T. cruzi* against 200 µM and 300 µM H_2_O_2_. We verified that the survival difference between overexpressing and control cells (transfected with the empty vector) was not due to a disparity in their doubling time, since they had similar growth rates when incubated in the absence of genotoxic treatment (Fig. 3C).

### Analysis of DNA lesions in *T. cruzi* nuclear and mitochondrial genomes

To examine how *T. cruzi* genome responds against genotoxic treatment, in particularly oxidative stress, we analyzed the generation and subsequent repair of DNA lesions in *T. cruzi* nuclear and mitochondrial genomes after treatment with H_2_O_2_ using a quantitative polymerase chain reaction (QPCR) assay described by Van Houten and colleagues [32].

We designed specific primers for the amplification of a 10 kb fragment of both nuclear and mitochondrial DNA of *T. cruzi*. Since this parasite mitochondrial DNA comprises around 25% of the total DNA content, we also designed specific primers for the amplification of a nuclear and mitochondrial 250 bp fragment, used to normalize changes in the proportion between nuclear and mitochondrial genomes. All primer sets amplified in a specific manner, with each primer pair producing only one band, verified by gel electrophoresis (data not shown).

**Figure 4:**
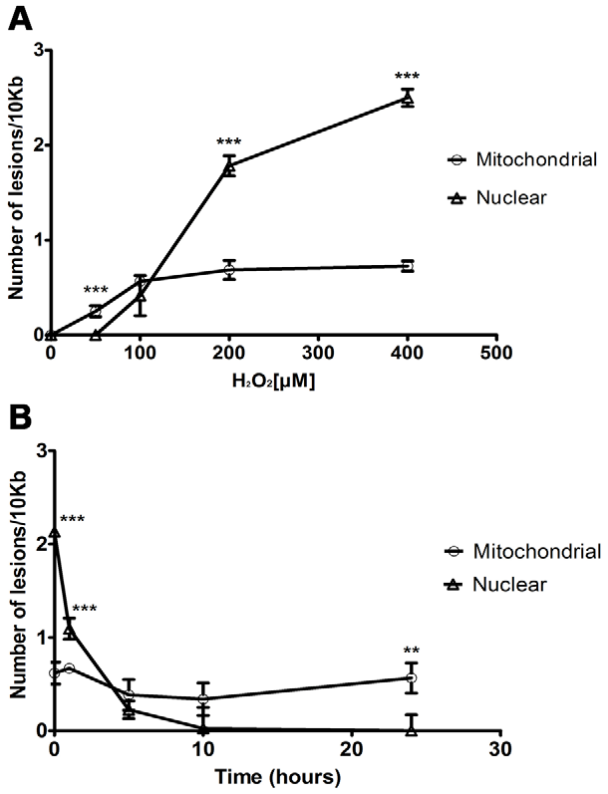
Analysis of DNA lesions in *T. cruzi* genome after treatment with H_2_O_2_. **A**) Nuclear and mitochondrial dose-responses after exposure to increasing H_2_O_2_ doses. Cells were treated for 15 min. **B**) Kinetics of damage and repair of the nuclear and mitochondrial fragments after exposure to 200 µM H_2_O_2_. Cells were treated for 15 min and allowed to recover for the times indicated. Data are expressed as the mean of two biological experiments. Error bars represent standard error of the mean. Statistical analysis used was unpaired *t* test. Mitochondrial DNA (**○**); Nuclear DNA (Δ). ***- P value<0,001; ** - P value<0,01.

Initially, a dose-response assay to see if treating the parasite with increasing H_2_O_2_ concentrations would result in a proportional increase of H_2_O_2_-induced DNA lesions was performed. At the lowest concentration tested (50 µM), H_2_O_2_ produced a low, but significant increase in lesions in mitochondrial, but not nuclear DNA. At concentrations of 100 µM or greater, a dose-dependent increase in nuclear lesions was observed. This is in contrast to mtDNA lesions which leveled off at concentrations of 100 µM or greater (Fig. 4A). To investigate the kinetics of *T. cruzi* DNA repair of H_2_O_2_-induced lesions, we analyzed the removal of those lesions during a period of 24 hours after the treatment. During that period, we also examined the parasite cell density to assure that the lesion reduction was not due to the replication of undamaged parasites or the death of the damaged ones, and we verified that the cell density did not change (data not shown). Fig. 4B shows that although treating *T. cruzi* with 200 µM H_2_O_2_ led to a higher lesion number within the nucleus, such lesions were totally repaired after 10 hours, whereas the mitochondrial lesions persisted after oxidative damage with little or no repair (Fig. 4B).

**Figure 5:**
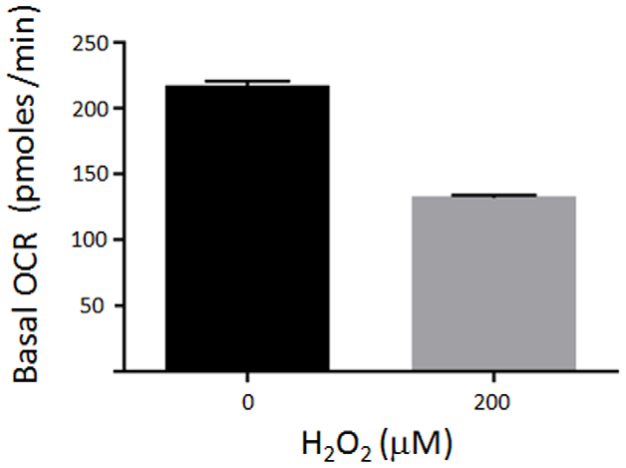
Mitochondrial function after H_2_O_2_ treatment. Analysis of the mitochondrial activity by measuring the basal oxygen consumption rate (OCR). Cells were treated with 0 µM and 200 µM H_2_O_2_ for 20 min and allowed to recover for 24 hours. Measures were done on the Seahorse Extracellular Flux Analyzer XF24. Results shown are representative of the mean of two independent experiments, performed in replicates of 3–4. The basal level of OXPHOS was calculated by the difference between the mean of rates 1 to 4 and the mean of rates 14 to 16. ** - P value<0,01.

The persistence of mtDNA lesions following H_2_O_2_ treatment was also observed in human cells by Van Houten and colleagues [32]. It was verified in this work that treatment with H_2_O_2_ could result in the loss of the mitochondrial function, which produced an increase in mtDNA lesions as a consequence of a second burst of oxidative species. To investigate whether the H_2_O_2_ treatment could cause a defect on *T. cruzi* mitochondrial function, we examined the oxygen consumption rate in parasites 24 hours after H_2_O_2_ treatment. Fig. 5 shows that *T. cruzi* cells presented a decrease rate of the oxygen consumption (a measure of mitochondrial function) after treatment with H_2_O_2_, which could explain the increase in mtDNA lesions after 24 hours.

**Figure 6:**
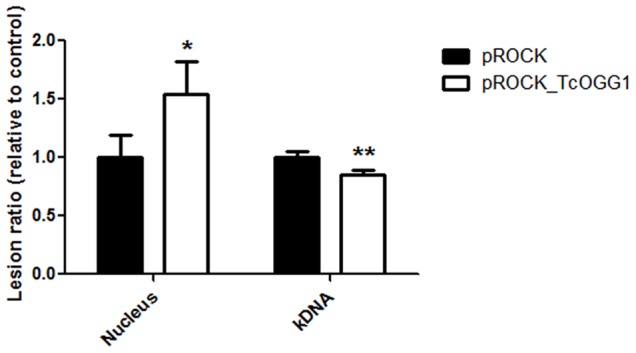
Analysis of DNA lesions in *TcOGG1*-overexpressor *T. cruzi*. Ratio between nuclear and mitochondrial DNA lesions from *TcOGG1*-overexpressing cells in comparison to control cells, after treatment with 200 µM H_2_O_2_. Both cell populations were treated for 20 min and allowed to recover for up to 24 hours. Data are expressed as the mean of two biological experiments. Error bars represent standard error of the mean. Statistical analysis used was unpaired *t* test. ** - P value<0,01; * - P value<0,1.

The AP sites and DNA strand breaks resulting from the removal of 8-oxoG by Ogg1, and 8-oxo-dG adducts by themselves, are absolute blocks to the progression of the PCR DNA polymerase [33], [34], [35]. Therefore, we expected that an elevated Ogg1 activity would give rise to a higher number of polymerase-blocking DNA lesions. To test this hypothesis we employed the QPCR technique to compare the number of DNA lesions between the *TcOGG1*-overexpressor and the control cells after treatment with H_2_O_2_. Our results show that after the treatment there were statistically more polymerase-blocking lesions in the nucleus of the *TcOGG1*-overexpressing cells than in the nucleus of control cells (Fig. 6). This result corroborates our hypothesis that the persistence of intermediate BER substrates is the main cause of the hypersensitivity to oxidative damage observed in *TcOGG1*-overexpressing cells. In contrast, the analysis of the mtDNA repair showed that control cells displayed more mtDNA lesions than overexpressing cells 24 hours after the treatment (Fig. 6).

### Overexpression of *TcOGG1* reduces the levels of 8-oxoG in the nucleus and in the mitochondrion of *T. cruzi*

To verify whether TcOgg1 recognizes 8-oxoG in the protozoan cellular context, we assessed the accumulation of 8-oxoG in the genome of *TcOGG1*-overexpressing and control cells. For that purpose we made use of avidin-conjugated FITC. Avidin is shown to bind 8-oxoG with high specificity and has been used to detect oxidative DNA damage in different cell types [36]. Thus, 8-oxoG levels can be inferred by the fluorescence intensity emitted by the nucleus and mitochondria of the FITC-avidin-treated parasites.

**Figure 7:**
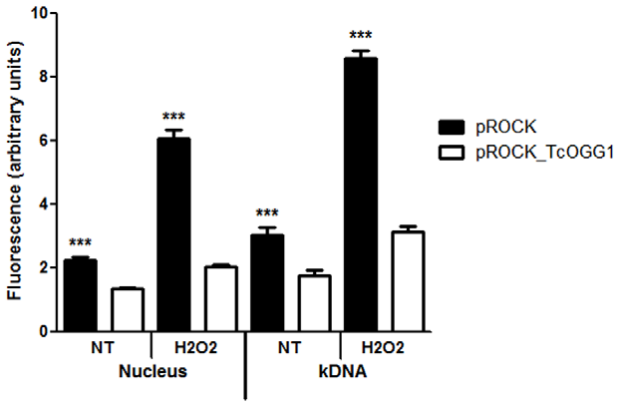
8-oxoguanine levels in nuclear and mitochondrial DNA of WT and *TcOGG1*-overexpressor *T. cruzi*, with or without H_2_O_2_ treatment. Cells were incubated with FITC-avidin, which binds to 8-oxoguanine. Slides containing stained parasites were visualized under a fluorescence microscope and fluorescence intensity was measured with ImageJ program. Graphics were plotted using the average of different experiments and statistical analyses used Mann Whitney test. NT: non-treated; kDNA: kinetoplast (mitochondrion) DNA. *** - P value<0,001.

As shown in Fig. 7, the levels of the 8-oxoG were lower in the nucleus of *TcOGG1*-overexpressing cells, when compared to control cells (p<0.001). This difference becomes more evident after treating the parasites for 20 minutes with 200 µM H_2_O_2_. Similar results were observed when mitochondria were examined, suggesting that TcOgg1 acts both in the nucleus and the mitochondrion of *T. cruzi*.

### *TcOGG1* is localized to a greater extent in the nucleus of *T. cruzi*

**Figure 8:**
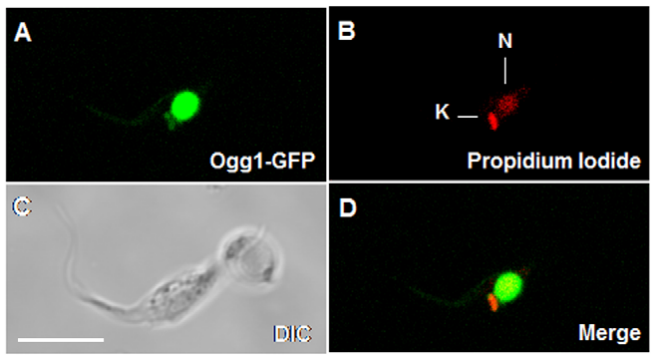
Subcellular localization of TcOgg1 in *T. cruzi*. CL Brener strain was transfected with pTREX_*TcOGG1*, resulting in the expression of TcOgg1 fused with GFP (Ogg1-GFP), which enabled the visualization of the protein under confocal microscope. DNA was stained with propidium iodide. Images obtained were analyzed with Zeiss LSM Image Browser software. N: nucleus; K: kinetoplast (mitochondrion); DIC: differential interference contrast. 5 uM scale bars is present in C.

The subcellular localization of TcOgg1 in *T. cruzi* was analyzed through the expression of this protein fused to GFP (**G**reen **F**luorescent **P**rotein) at its C-terminus, using the construction pTREX_*TcOGG1*-*GFP* [30]. As shown in Fig. 8, the Ogg1-GFP fusion protein was predominantly localized in the nucleus of the parasite, but was also found in the kinetoplast region of the mitochondrion. This cellular localization is in agreement with the results showing decreased levels of 8-oxoG in both organelles (Fig. 7).

## Discussion

Data presented in this study indicate that we have cloned and characterized the *T. cruzi* gene encoding 8-oxoguanine DNA glycosylase (OGG1). Furthermore this study revealed that unlike mammalian cells, hydrogen peroxide induced high levels of nuclear DNA damage as compared to mtDNA damage.

There are two putative *OGG1* gene sequences in the CL Brener genome database (the sequenced *T. cruzi* strain) [20]. CL Brener is a hybrid strain, thus it is common to find two different alleles for a gene in its genome. *TcOGG1* alleles present only minor differences (Fig. S1). Indeed, independent mutation assays performed in yeast expressing *TcOGG1*_A or *TcOGG1*_B showed that both alleles complement in the same extent the hypermutator phenotype of the *OGG1*-deficient yeast strain.

The heterologous functional complementation assay, used in this work to study *TcOGG1* role, is an useful technique to study eukaryotic DNA repair genes [37]. We have chosen *S. cerevisiae* as a heterologous system to study *TcOGG1* because the expression of this gene was toxic to *E. coli* (Figure S2 A–C). Similar toxicity was not found in literature, since *OGG1* from different organisms (e.g., *S. cerevisiae*, *A. thaliana*, *H. sapiens*) were expressed in bacteria lacking its *OGG1* ortholog, *fpg* gene [9], [16], [18]. Furthermore, several *T. cruzi* genes (such as *TcPOLK*, *TcPOLH*, *TcMSH2*, *TcPOLB* and *TcPOLB-PAK*), were expressed in *E. coli*, suggesting that this phenotype is *OGG1*-specific [28], [30], [31], [38], [39]. We observed that the putative *T. cruzi OGG1* suppresses the spontaneous mutator phenotype of *OGG1*-deficient yeast (Fig. 2A and 2C), which suggests that this *T. cruzi* gene presents the expected 8-oxoguanine DNA glycosylase activity. Furthermore, this result is consistent with the previous observation that *fpg* from *E. coli* reverses the high mutation frequency of CD138 yeast strain [40].

We have seen that a *TcOGG1*-overexpressing CL Brener lineage is viable and grows normally in non-stressed conditions (Fig. 3C). In oxidative stressed conditions, the overexpressor cells show reduced survival (Fig. 3B), which could be explained by BER imbalance. The excess of Ogg1 activity might result in strand breaks, blocking cell transcription and replication processes, causing cytotoxicity. This excess of strand breaks could also lead to energy depletion due to over activity of PARP, which causes intense reduction of NAD^+^ levels, decreasing cell viability [41]. It has been reported that the overexpression of glycosylases Mag1 and Tag sensitized *E. coli* and yeast to killing by the alkylating agent methyl methanesulfonate (MMS) [42], [43] and generates a mutator phenotype in yeast [42]. In the same way, the overexpression of Mag1 in mammalian cells is also deleterious [44]. Also, mammalian cells overexpressing Ogg1 show increased lethality and enhanced double strand breaks formation after treatment with DNA damaging agents, when compared to control cells [45], [46], [47].

To test our hypothesis that the increased H_2_O_2_ sensibility of *TcOGG1*-overexpressor cells is a result of an excessive generation of cytotoxic lesions, we measured DNA damage using the QPCR technique [48]. We have used this methodology previously in order to quantify DNA lesions in different organisms such as *Caenorhabditis elegans* [49] and mammal cells [50]. When we assayed the DNA repair of *T. cruzi* wild type cells after treatment with H_2_O_2_, we found unexpectedly that H_2_O_2_ treatment leads to high levels of DNA damage in the nucleus and low DNA damage in the mitochondria of *T. cruzi* (Fig. 4 A). This contrasts to what is observed in mammal cells, which, after H_2_O_2_ treatment, present DNA damage only in the mitochondria when QPCR methodology is used [51]. The lack of nuclear DNA lesions in mammal cells is explained by the low availability of iron and copper atoms in this organelle, which might otherwise contribute to the Fenton reaction and generate highly reactive hydroxyl radicals [51]. Thus, the unexpected increase in DNA lesions in *T. cruzi* nucleus suggest increased levels of iron and copper in this parasite's compartment. In addition, *T. cruzi* enzymes involved in ROS neutralization are present only in the cytoplasm, glycosomes and mitochondria [52]. Therefore, the absence of these enzymes in *T. cruzi* nucleus might be one of the causes of this discrepancy between the levels of nuclear and mitochondrial DNA lesions.

In contrast to the nuclear DNA lesions, which are efficiently removed (Fig. 4B), mtDNA lesions are not repaired, even though H_2_O_2_ treatment causes relatively low levels of mitochondrial DNA damage. In fact, the number of mtDNA lesions increased during the recovery period, when the agent was removed from the media (Fig. 4B). ROS interaction with lipids and proteins of mitochondria membrane can cause loss of membrane potential, compromising the efficiency of cell respiration. This in turn can further give rise to a second burst of ROS that can result in more DNA damage [51]. Our findings showing that H_2_O_2_ treatment decreases *T. cruzi* respiration (Fig. 6) corroborate the idea that the subsequent increase of mtDNA lesions could be driven by a second burst of ROS, caused by a reduction in mitochondrial function.

The QPCR results obtained herein are in agreement with a recently published article that investigated *T. cruzi* BER pathway [53]. In that work, the levels of nuclear and mitochondrial DNA damage generated by H_2_O_2_ were similar to the DNA damage profile verified by our group.

The observed dual localization of TcOgg1 (Fig. 8) was expected due to the presence of a nuclear and mitochondrial targeting signals in its protein sequence (Fig. S1). These results are consistent with the nuclear and mitochondrial localization of Ogg1 described in previous reports [54], [55], [56], [57]. Our measurements of 8-oxoG levels also reinforce the fact that TcOgg1 acts in both organelles.

Our QPCR results regarding the nuclear DNA supported the assumption that *TcOGG1*-overexpression increases the sensitivity to H_2_O_2_ as a result of excess in AP sites and/or strand brakes, since the level of DNA lesions in nucleus are higher in *TcOGG1*-overexpressing cells (Fig. 5A). In contrast, *TcOGG1*-overexpressing cells have less mitochondrial DNA damage than control cells, after treatment with H_2_O_2_ (Fig. 5B). We speculate that the overexpression of TcOgg1 did not increase mtDNA lesions because it does not lead to uncoupled BER in the mitochondria.. The cellular localization experiment supports this idea, since it showed that overexpressing TcOgg1-GFP leads to an intense signal in the nucleus but only generates a subtle signal in the mitochondria (Fig. 8). In addition to that, previous work have shown that *T. cruzi* mitochondria contain relatively high levels of DNA polymerase β [39] and DNA ligases kα and kβ [58]. Once the AP site generated by the glycosylase is correctly processed (which can be performed by OGG1 lyase activity), Pol β is capable of filling the gap. On the other hand, the further ligation steps might be carried out by DNA ligase kα or DNA ligase kβ. Therefore, it would be possible that the overexpression of TcOgg1 is producing a bottleneck in the nuclear BER pathway (with increased levels of AP sites and/or strand brakes), but not in the mitochondrial BER.

The fact that *TcOGG1*-overexpression did not increase mtDNA lesions after H_2_O_2_ treatment could also be a consequence of the simultaneous activation of another DNA repair pathway. Thomas and colleagues suggest that *T. cruzi* mtDNA display high rates of mitochondrial DNA recombination [59]. In addition, previous work from our research group corroborates the idea that *T. cruzi* uses DNA recombination for repairing its mitochondrial DNA [30]. Thus, it is possible that AP sites and/or strand breaks generated by enhanced glycosylase activity could trigger recombination repair, which in turn would reduce the number of cytotoxic lesions detected by our methodology.

These data indicate that repair pathways that work to correct oxidative lesions in *T. cruzi* are important for the biology of the parasite. Future experiments must be performed to determine the extent by which *T. cruzi* can deal with other types of oxidative lesions. To this end we are currently characterizing the other components of the GO system (MutT and MutY orthologues).

## Materials and Methods

### Alignment

Sequence alignments and motif analyses were performed using the Multalin [60] and Boxshade 3.21 (www.ch.embnet.org/) interfaces. Protein-targeting signals were predicted using MITOPROT [61], NucPred [62], ESLpred [63] and SubLoc [64]. GenBank accession numbers of the Ogg1 sequences used in this work: *Trypanosoma cruzi* (*TcOGG1*_A (accession number XP_821796.1); *TcOGG1*_B (accession number XP_804159.1), *Trypanosoma brucei* (XP_844398.1), *Leishmania major* (XP_001686315.1), *Arabidopsis thaliana* (CAC19363.1), *Mus musculus* (AAB94512.1), *Homo sapiens* (AAB81132.1) and *Saccharomyces cerevisiae* (AAC49312.1).

### *E. coli*

#### 1. Strains and plasmids

*E. coli* strains used in this article were DH5α (*sup*E44, *lac*U169, *hsd*R17, *rec*A1, *end*A1, *gyr*A96, *thi*1, *rel*A1) [65], AB1157 (*F*- *thr-1, leu*B6, *thi-1, arg*E3, *his*-G4, *Δ(gpt-pro*A*)*, 62*lac*Y1, *gal*K2, *xyl*-5, *ara*-14, *rpsL*31, *kdg*K51 *mtl*-1, *txs*-33, *sup*E44 (str^R^) *rfb*D1) [66] and its derivative BH20 (as AB1157, *fpg1*::Tn5 (kan^R^)) [67]. Bacteria were grown at 37°C, with agitation (180 rpm), in 2×YT medium containing ampicillin (100 µg mL^−1^) and supplemented with kanamycin (10 µg mL^−1^) or streptomycin (10 µg mL^−1^), depending on the bacterial strain. *TcOGG1* was amplified from the genomic DNA of CL Brener strain using the primers TcOGG.Xba-F (5′-TCTAGAATGCACGCGTGGTATGCG-3′) and TcOGG.Hind-R (5′- AAGCTTTCAGTTGTCTCTTTGCCC-3′). The amplification product was cloned into pMAL-c2G (NE BioLabs Inc.) using *Xba*I and *Hind*III restriction sites in order to produce pMAL_*TcOGG1*. Bacteria were transformed by electroporation [68].

#### 2. Growth curves

*E. coli* cells carrying the empty vector pMALc2G or pMAL_*TcOGG1* were previously grown for 16 hours. After this, they were diluted to OD_600_ = 0.02 and incubated in the presence (0.1 mM) or in the absence of IPTG. The bacterial growth was recorded by sampling the cultures at each 60 min, measuring the absorbance at λ = 600 nm with a spectrophotometer (Shimadzu, UV-1203). Each experiment was performed in triplicates and the absorbance values were plotted as function of time.

### *Saccharomyces cerevisiae*

#### 1. Strains and plasmids

*S. cerevisiae* strains used were FF18733 (*MATa*, *his7*, *leu2*, *lys1*, *ura3*, *strp1*) [26] and its derivative CD138 (*MATa*, *his7*, *leu2*, *lys1*, *ura3*, *ogg1*::*TRP1*) [25]. *TcOGG1* was amplified from CL Brener strain, using the primers TcOGG.Bam-F (5′-GGATCCATGCACGCGTGGTATG-3′) and TcOGG.Sac-R (5′-GAGCTCTCAGTTGTCTCTTTGCC-3′). The amplification product was inserted into pYEDP (kindly donated by Dr. Francisco Nóbrega, UNIVAP, São Paulo) using *Sac*I and *Bam*HI restriction sites in order to produce pYEDP_*TcOGG1*. Yeast strains were transformed using the acetate lithium treatment [69].

#### 2. Spontaneous mutation frequencies

*S. cerevisiae* cells were grown to the stationary phase in liquid YPD medium, at 30°C with agitation (240 rpm). The cultures were plated in SD agar plates without lysine, containing glucose or galactose, in a cell density of approximately 5×10^5^ cells mL^−1^. All the plates were supplemented with histidine (20 µg mL^−1^), leucine (100 µg mL^−1^) and tryptophan (20 µg mL^−1^). The plates were incubated at 30°C for 3 days (glucose) or for 5 days (galactose) and then the number of Lys^+^ revertant colonies was counted.

### *T. cruzi*

#### 1. Parasite growth and transfection

Epimastigote forms of the CL Brener strain of *T. cruzi* were grown at 28°C in liver infusion tryptose (LIT) medium (pH 7.3) supplemented with 10% heat-inactivated fetal bovine serum, 100 U mL^−1^ penicillin and 100 µg mL^−1^ streptomycin as described [70].

The vector pROCK_TcOGG1 was constructed amplifying *TcOGG1* with the primers TcOGG.Xba-F and TcOGG.Xho-R (5′-CTCGAGTCAGTTGTCTCTTTGCCC-3′) and cloning the amplification product into *Xba*I and *Xho*I restriction sites of pROCK_HYGRO. The vector pTREX_TcOGG1-GFP was constructed amplifying *TcOGG1* with the primers TcOGG.Xba-F and TcOGG.Mfe-R (5′-CAATTGGTTGTCTCTTTGCCCTCTTCG-3′) and inserting the amplification product into *Xba*I and *Mfe*I sites of pTREX-GFP. The parasite transfection was performed by electroporation according to a previously described protocol [27]. Transfected parasites containing stably incorporated pROCK_TcOGG1 expression vector were selected after 6 weeks of culturing in the presence of Hygromycin (200 mg mL^−1^).

#### 2. Northern blot analysis

For Northern blot analysis, 15 µg of total RNA was size fractionated in 1.2% agarose gel containing 5% formaldehyde, blotted onto a Hybond-N+ membrane (GE healthcare) by capillary transfer, and cross-linked by UV irradiation. A DNA probe for TcOGG1 gene was amplified by PCR, gel purified and labeled with [α-^32^-P]-dCTP using the Megaprime TM DNA labeling protocol from GE Healthcare. The membrane was hybridized with 2X SSC/0.1% SDS at 60°C, as previously described [71].

#### 3. Growth and survival curves

Parasite cultures containing initially 5×10^6^ parasites mL^−1^ were incubated at 28°C for approximately 5 days. The cell growth was monitored by counting parasites after 0, 29, 47, 76 and 106 hours of incubation. Cells were counted in a cytometric chamber using erythrosine vital stain to differentiate living and dead cells. For testing resistance to H_2_O_2_, parasite cultures containing 5×10^6^ parasites mL^−1^ were treated with 0, 100, 200 or 300 µM H_2_O_2_. After incubation for 3 days, cell number was measured by counting as described above. The results were expressed as percentage of growth when compared to untreated cultures. Experiments were performed in triplicate.

#### 4. Analysis of DNA lesions after H_2_O_2_ treatment using quantitative PCR assay (QPCR)

Parasite cultures containing 1×10^7^ cells mL^−1^ were harvested by centrifugation at 3000 *g* for 10 min. The supernatant medium (conditioned medium) was saved for later use and the cells were re-suspended in PBS. In the dose-response assay, cells were treated by incubating the parasites with 0, 50, 100, 200 or 400 µM H_2_O_2_ for 15 min. The DNA repair assay was performed by incubating parasites with 200 µM H_2_O_2_ for 15 min or 20 min, as indicated. After the treatment, cells were harvested immediately or allowed to recover for up to 24 h (in this case, in the original conditioned medium). The DNA extraction, quantification, QPCR amplification and result analyses were conducted as reported by Santos *et al.* (2006) [48]. The QPCR assay is performed comparing the amplification of the DNA from a treated sample with the amplification of the undamaged control. Specific primers were used to amplify large and small fragments of the nuclear and mitochondrial DNA. The large nuclear fragment was amplified using the forward primer QPCRNuc2F (5′-GCACACGGCTGCGAGTGACCATTCAACTTT-3′) and the reverse primer QPCRNuc2R (5′-CCTCGCACATTTCTACCTTGTCCTTCAATGCCTGC-3′). The small nuclear fragment was amplified employing the internal primer QPCRNuc2Int (5′-tcgagcaagctgacactcgatgcaaccaaag-3′) and the reverse primer QPCRNuc2R. The large mitochondrial fragment was amplified using the forward primer QPCRMitF (5′-TTTTATTTGGGGGAGAACGGAGCG-3′) and the reverse primer QPCRMitR (5′-TTGAAACTGCTTTCCCCAAACGCC-3′). The small mitochondrial fragment was amplified with the internal primer QPCRMitInt (5′-CGCTCTGCCCCCATAAAAAACCTT-3′). Because the probability of introducing a lesion in a short segment is very low, the small fragment (250 pb) is used to normalize the amplification results obtained with the large fragments (10 kb), which eliminates the bias of changes in the proportion between nuclear and mitochondrial genomes. The normalized amplification of treated samples was then compared with controls, and the relative amplification was calculated. These values were next used to estimate the average number of lesions per 10 kb of the genome, using a Poisson distribution. The final results are the mean of two sets of PCR for each target gene of at least 2 biological experiments.

#### 5. Oxygen consumption assay

Cells were treated with 200 µM H_2_O_2_ as described previously in this section. The analysis of the oxygen consumption rate (OCR) was performed as described by Qian and Van Houten (2010) [72]. The OCR was measured in real-time using a Seahorse Bioscience XF24 Extracellular Flux Analyzer (Billerica). After treatment with H_2_O_2_, cells were seeded into XF24-well microplates plates (5×10^7^ cells per well) coated with BD Cell-Tak Cell Adhesive, and then washed and incubated in unbuffered DMEM (pH 7.4) supplemented with GlutaMax-1 (2 mM), glucose (25 mM), sodium pyruvate (1 mM), sodium chloride (32 mM) and phenol red for 1 h at 37°C without CO_2_. The basal OCR was measured and the results were analyzed using the algorithm described by Gerencser *et al.* [73].

#### 6. Measurement of 8-oxoguanine accumulation

A protocol adapted from Struthers *et al.* (1998) [36] was used to assess the 8-oxoG accumulation in *T. cruzi* DNA strains. Epimastigotes were harvested, washed with PBS and incubated at 28°C, for 20 min, in the presence of 200 µM H_2_O_2_. After the genotoxic exposure, cells were washed with PBS and fixed with 4% paraformaldehyde for 20 min at 4°C. Cells were washed again and ressuspended in PBS to a final concentration of 6×10^8^ cells mL^−1^. Aliquots of the cell suspension were distributed onto 8-wells chambered-slides. After 1 h incubation at 4°C for adhesion, cells were permeabilized with 0.2% Triton X-100, treated with 100 µg mL^−1^ RNAse A and incubated with FITC-conjugated avidin (5 µg mL^−1^ final concentration) for 1 h at room temperature in the dark. After washing with PBS and mounted with a solution of 9∶1 Glycerol∶Tris-HCl pH 9.0, the slides were visualized on a fluorescence microscope. The fluorescence intensity was averaged with the ImageJ program (http://rsbweb.nih.gov/ij/) and plotted as fluorescence arbitrary units (average fluorescence intensity measured in 100 cells after subtracting the average background intensity).

#### 7. Confocal microscopy

Epimastigote forms of *T. cruzi* were transfected with the pTREX vector encoding GFP or *TcOGG1* fused to GFP. Transfected parasites were harvested 24 h after electroporation, washed once in PBS and centrifuged at 3000 *g* for 10 min. Cell pellets were re-suspended in PBS such that the sample was concentrated 100-fold and then fixed in 4% paraformaldehyde for 15 min at 4°C. The paraformaldehyde-treated cells were then washed with PBS, treated with 0.1 mg mL^−1^ RNAse for 15 min at 37°C, and then incubated with 20 µg mL^−1^ propidium iodide for 15 min to stain DNA. Epimastigotes were washed in PBS and placed on slides. Images were captured with a Zeiss LSM 510 META microscope using 488 and 543 nm lasers for probe excitation, with the pinhole aperture adjusted to 1 airy unit. The images were analyzed with Zeiss LSM Image Browser software.

### Graphics and statistical analysis

All graphics and statistical analyses were performed using the GraphPad Prism 5.0 software.

## Supporting Information

Figure S1 **Alignment of the predicted products of the two** ***TcOGG1*** **alleles,** ***TcOGG1*** **_A and** ***TcOGG1*** **_B.** Residues shaded in black are identical. Amino acids shaded in grey are functionally similar. The region highlighted by the symbol “□” indicates the predicted mitochondrial targeting sequence and the region marked with the symbol “▪” represents nuclear localization signal. Residues enclosed by the box belong to the HhH-G/PD motif.
(TIF)

Figure S2 **TcOgg1 toxicity in** ***E. coli*** **.** **A**) Growth of DH5α *E. coli* on agar plates. Bacterial cells were plated on agar plates containing ampicillin (Amp) or ampicillin+IPTG (Amp+IPTG; expression of the gene inserted into the vector). Different numbers refer to different clones. pMAL-c2G: empty vector. **B and C**) Growth curves from AB1157 (*fpg*+) and BH20 (*fpg*−) *E. coli*, respectively. Bacteria were grown with or without IPTG and had their ODs read in certain time intervals. The curves are the average of three independent experiments and the bars represent SEM. pMAL (▴); pMAL+IPTG (▾); pMAL_*TcOGG1* (•); pMAL_*TcOGG1*+IPTG (▪).
(TIF)

Figure S3 **Heterologous complementation assay with FF18733 (WT) and CD138 (** ***ogg1*** **-) yeast –** ***TcOGG1_A*** **.** . **A and B**) Quantitative analysis. Mutants obtained in the assay were counted, originating Figures S3A–B. Fig. S3 A shows the results for glucose, whereas Fig. S3 B displays the results for galactose. The graphics were plotted using median and the statistical analysis used was Kruskal-Wallis test (One way ANOVA). FF.pYEDP (•); CD.pYEDP (▪); CD.pYEDP_*TcOGG1* (▴). ***- P value<0,001; ** - P value<0,01.
(TIF)
