# Supplementary figures and images for: Functional Characterization of 8-Oxoguanine DNA Glycosylase of Trypanosoma cruzi
Source: PLoS One. 2012 Aug 2;7(8):e42484. doi: 10.1371/journal.pone.0042484 (PMC3411635; doi:10.1371/journal.pone.0042484)

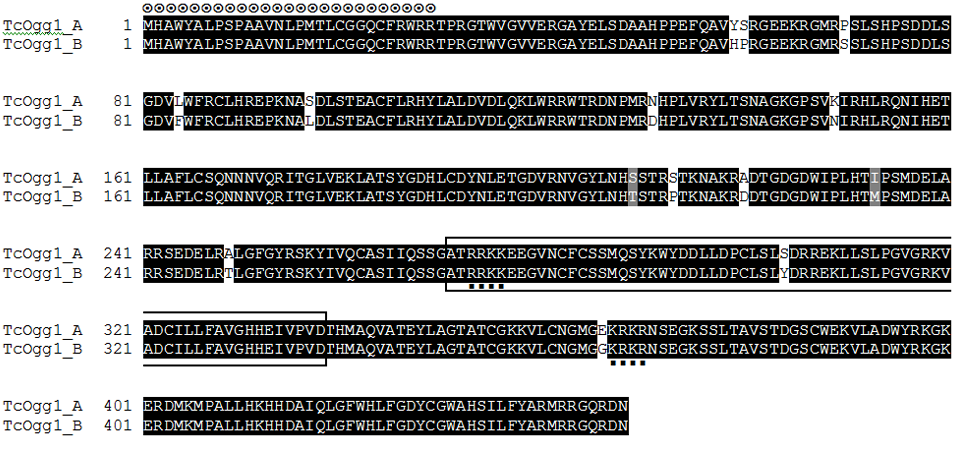

Supplement: Figure S1 — Alignment of the predicted products of the two TcOGG1 alleles, TcOGG1 _A and TcOGG1 _B. Residues shaded in black are identical. Amino acids shaded in grey are functionally similar. The region highlighted by the symbol “□” indicates the predicted mitochondrial targeting sequence and the region marked with the symbol “▪” represents nuclear localization signal. Residues enclosed by the box belong to the HhH-G/PD motif. (TIF) [file pone.0042484.s001.tif]

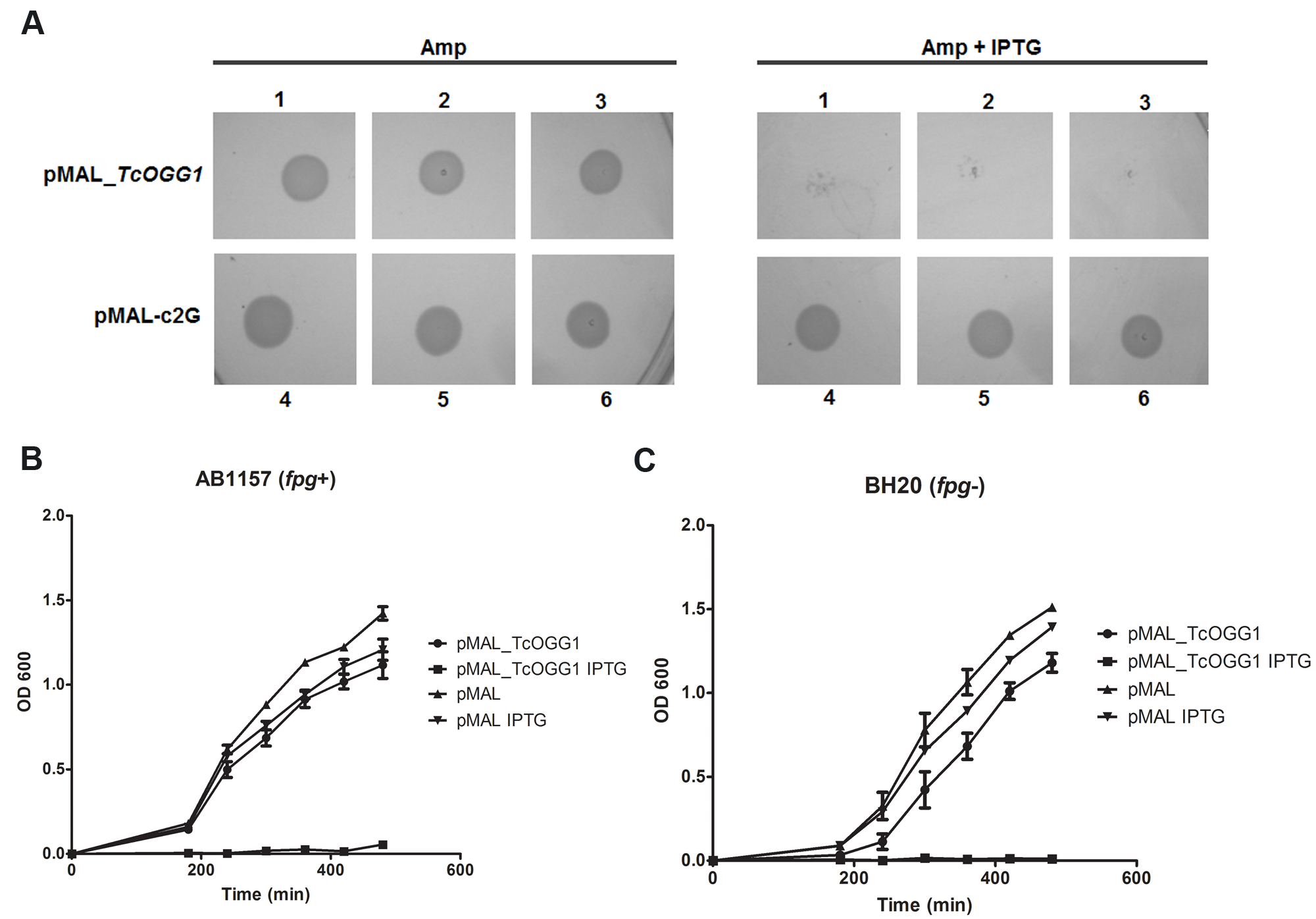

Supplement: Figure S2 — TcOgg1 toxicity in E. coli . A) Growth of DH5α E. coli on agar plates. Bacterial cells were plated on agar plates containing ampicillin (Amp) or ampicillin+IPTG (Amp+IPTG; expression of the gene inserted into the vector). Different numbers refer to different clones. pMAL-c2G: empty vector. B and C) Growth curves from AB1157 (fpg+) and BH20 (fpg−) E. coli, respectively. Bacteria were grown with or without IPTG and had their ODs read in certain time intervals. The curves are the average of three independent experiments and the bars represent SEM. pMAL (▴); pMAL+IPTG (▾); pMAL_TcOGG1 (•); pMAL_TcOGG1+IPTG (▪). (TIF) [file pone.0042484.s002.tif]

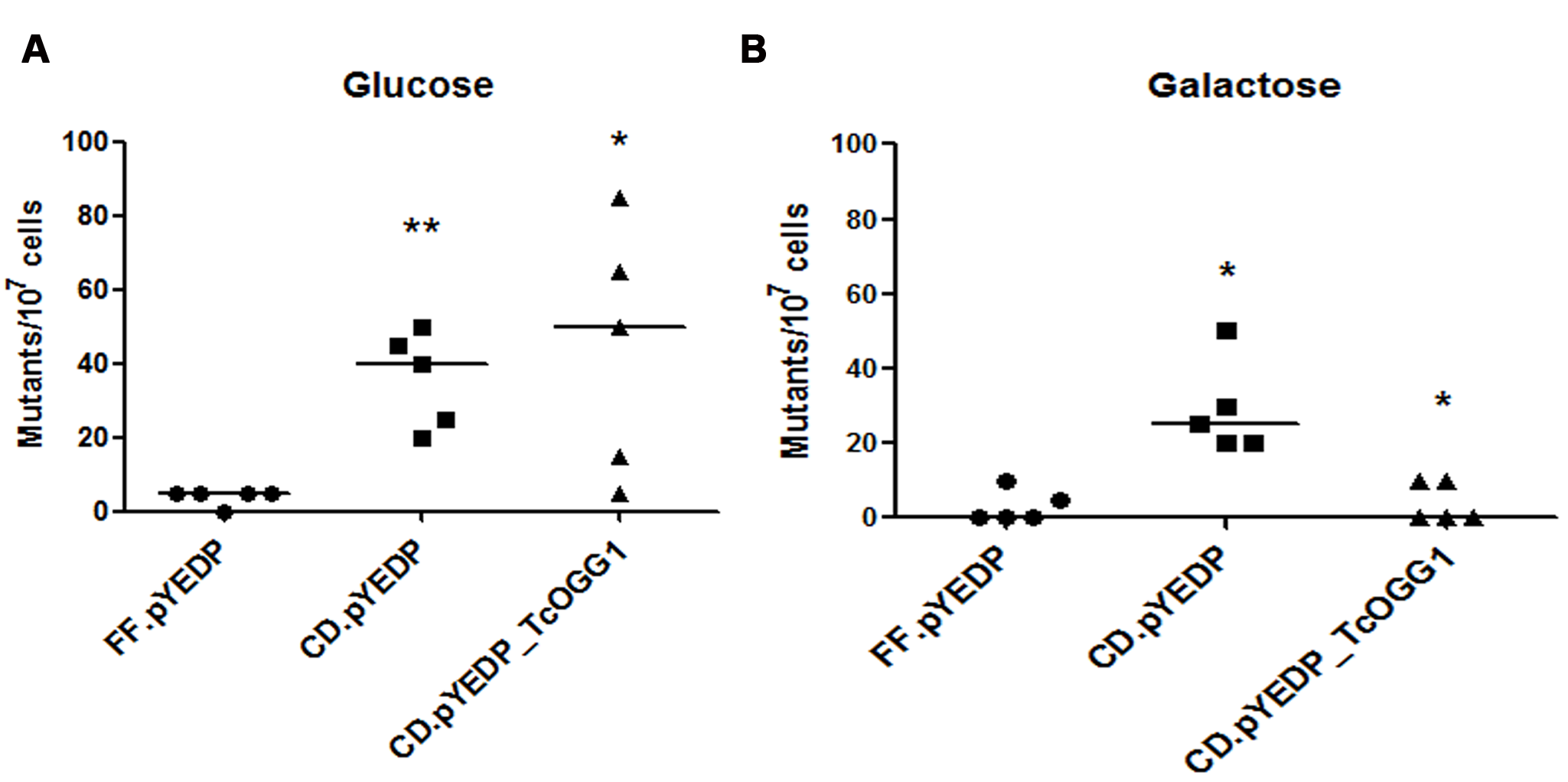

Supplement: Figure S3 — Heterologous complementation assay with FF18733 (WT) and CD138 ( ogg1 -) yeast – TcOGG1_A . . A and B) Quantitative analysis. Mutants obtained in the assay were counted, originating Figures S3A–B. Fig. S3 A shows the results for glucose, whereas Fig. S3 B displays the results for galactose. The graphics were plotted using median and the statistical analysis used was Kruskal-Wallis test (One way ANOVA). FF.pYEDP (•); CD.pYEDP (▪); CD.pYEDP_TcOGG1 (▴). ***- P value<0,001; ** - P value<0,01. (TIF) [file pone.0042484.s003.tif]
